# Responses to climatic and pathogen threats differ in biodynamic and conventional vines

**DOI:** 10.1038/s41598-018-35305-7

**Published:** 2018-11-15

**Authors:** Isabelle Soustre-Gacougnolle, Marc Lollier, Carine Schmitt, Mireille Perrin, Estelle Buvens, Jean-François Lallemand, Mélanie Mermet, Mélanie Henaux, Christelle Thibault-Carpentier, Doulaye Dembelé, Damien Steyer, Céline Clayeux, Anne Moneyron, Jean E. Masson

**Affiliations:** 1 SVQV, Université de Strasbourg, INRA, 28 route de Herrlisheim 68021, BP, 20507 Colmar, France; 2 LVBE, EA3991, Université de Haute Alsace, 33 rue de Herrlisheim, 68000 Colmar, France; 3 GIEE, 1 rue de Rouffach 68250 Westhalten; c/o Jean-Francois Lallemand, Colmar, France; 4 GenomEast Platform, Institut de Génétique et de Biologie Moléculaire et Cellulaire (IGBMC), 1 rue Laurent Fries/BP 10142/, 67404 Illkirch, France; 5 TWISTAROMA; Université de Strasbourg, Faculté de Pharmacie, 74 route du Rhin, 67400 Illkirch, France; 6 Tuque Rouge, 47500 Cuzorn, Colmar, France

## Abstract

Viticulture is of high socio-economic importance; however, its prevalent practices severely impact the environment and human health, and criticisms from society are raising. Vine managements systems are further challenged by climatic changes. Of the 8 million hectares grown worldwide, conventional and organic practices cover 90% and 9% of acreage, respectively. Biodynamic cultivation accounts for 1%. Although economic success combined with low environmental impact is widely claimed by biodynamic winegrowers from California, to South Africa, and France, this practice is still controversial in viticulture and scientific communities. To rethink the situation, we encouraged stakeholders to confront conventional and biodynamic paradigms in a Participative-Action-Research. Co-designed questions were followed up by holistic comparison of conventional and biodynamic vineyard managements. Here we show that the amplitude of plant responses to climatic threats was higher in biodynamic than conventional management. The same stood true for seasonal trends and pathogens attacks. This was associated with higher expression of silencing and immunity genes, and higher anti-oxidative and anti-fungal secondary metabolite levels. This suggests that sustainability of biodynamic practices probably relies on fine molecular regulations. Such knowledge should contribute to resolving disagreements between stakeholders and help designing the awaited sustainable viticulture at large.

## Introduction

Of the 8 million hectares devoted to viticulture worldwide, conventional and organic practices cover 90% and 9% of acreage, respectively. Biodynamic cultivation accounts for only ca. 1% (http://www.demeter.net/contacts-di), and this practice is still controversial. Studies on biodynamic or/and organic cultivation focused on economy and soil composition^1^, on soil structure, soil biodiversity and microbiological activity^2^, and on fertilizer effects^3^. Studies of grape yield and quality^4,5^, microbial communities in grapes and leaves^6,7^, and wine biochemistry^8^ have also been reported. However, the results have not resolved the controversy^9^ about the ‘anthroposophical paradigm’^10^ at the heart of biodynamics practice. To date, the dissenting viticulture communities have not reached a collective plan to reduce the impact of viticulture practices on the environment and human health^11,12^. We have tentatively addressed this problem holistically, bringing together the different stakeholders in a Participative-Action-Research^13^. The workshops highlighted the dissensus among biodynamic and conventional winegrowers, as well as among the non-government organizations Alsace Nature/France Nature Environment, viticulture advisors, technicians, and research scientists. The dissensus stems from lack of distinct proof of the claims made, and from epistemic conflicts^13,14^. Relying on a collective epistemology^13^, the group arrived at the most sensitive and controversial issues. Biodynamic winegrowers lacked experimental evidence for their claim that their practices stimulate plant defense mechanisms. The conventional winegrowers have shown that synthetic pesticides deter pathogens (albeit with a high environmental impact) but there were no data on possible effects on plant defenses. Focusing on plant responses to pathogen and climatic threats, we chose a holistic approach to compare the consequences of the differing practices. We chose Pinot Noir as it is used worldwide for wines and champagne, its genome has been fully sequenced^15^, and its sensitivity to climatic disorders is well documented^16,17^. Our trial consisted of 14 plots of Pinot Noir vines grafted onto the SO_4_ rootstock that had been grown under conventional management (8 winegrowers, 8 plots of 21,413 m^2^) or biodynamic management (3 winegrowers, 6 plots of 9,756 m^2^) for more than 20 years in the same climatic conditions (Fig. S1). Over a 4-year period, we monitored vine management, plant physiology, and the levels of infection with virus, downy mildew, and powdery mildew. For plant defense responses, we analyzed secondary metabolite content as well as steady-state mRNA levels of 30 immunity and silencing genes.

## Results and Discussion

### Soils, climatic conditions and vine management

When comparing soils in conventional/biodynamic management, after chemical and physical analysis, values were higher for pH, Calcium and soil components above 2 mm in conventional, and higher for Manganese and penetrometer assays in Biodynamic, suggesting steeper horizons (Mann-Whitney at *P ≤ 0.05; Table S1). Still, there was no clear-cut picture between soils from biodynamic and conventional plots, in contrast to other studies^1,2,4^. The conclusions of the latter authors, however, were based on additional biochemical and biological parameters, and here, we cannot exclude that further analysis, such as in microbiology, may reveal differences as illustrated in organic and conventional management^7^.

**Figure 1:**
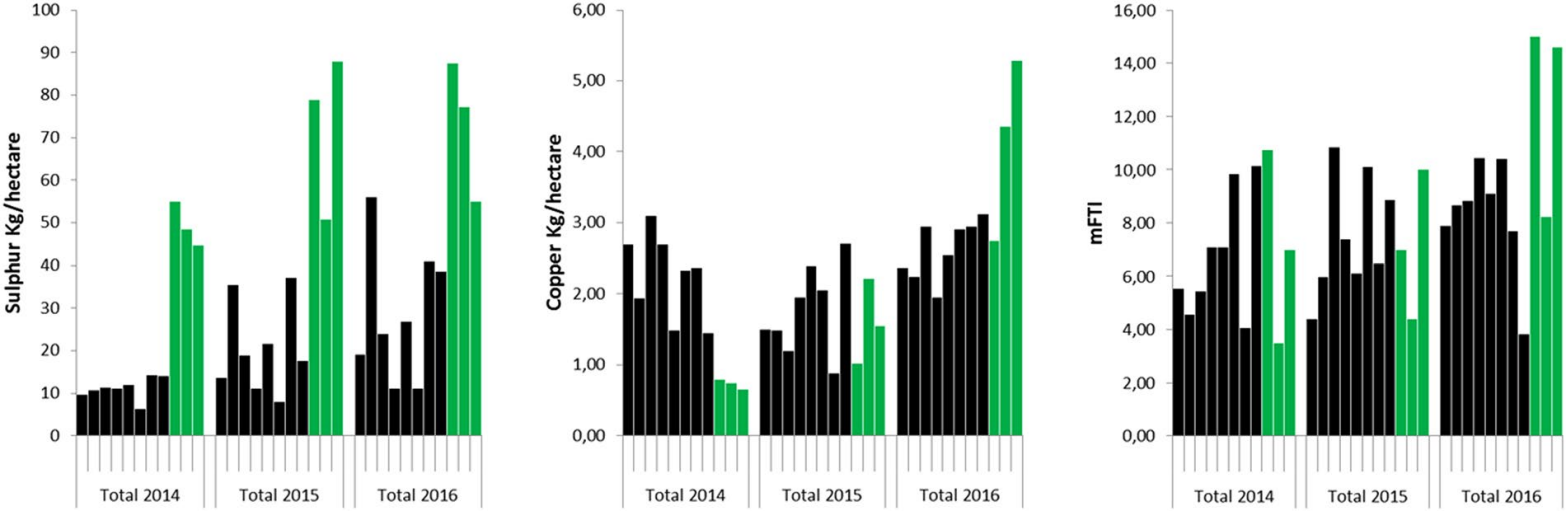
Characterization of conventional and biodynamic viticulture-practices. Data for each month were collected across 2014–2016. Index (modified Treatment Frequency Index) mTFI = product dose used x field surface sprayed/recommended dose x full field surface. Doses are recommended by supplier or/and government for synthetic fungicides in conventional practices (CABRIO TOP, CANTUS, CYFLODIUM, DIAZOLE TL, ELECTIS, ELECTIS BLEU, EMENDO V, HOGGAR, KENKIO, KESIS, MILDICUT, NATIVO, PANTHEOS, PERGADO MZ PEPITE, PROFILER, SWITCH, TALIUS, VIVANDO, YSAYO, (black). Biodynamic composts and preparations (cow manure 500, 500 P, finely ground silica (501), decoctions of nettle (504), willow, horsetail, valerian, and lemon oil, according to Biodynamic guide (green). Copper and Sulfur sprayings in conventional and biodynamic practices (black and green, respectively). Sum of values/year with a bar for each winegrower, black and green for conventional and biodynamic, respectively. Full treatments on vineyards = mTFI + Copper + Sulphur for each time-period.

We characterized pest management practices after interviews with winegrowers^13^. All winegrowers in the study relied on copper and Sulfur treatments. In addition, conventional viticulture employed synthetic fungicides, whereas biodynamic cultures were complemented with preparations such as cow manure (500, 500 P), minerals such as finely ground silica (501), decoctions of nettle (504), willow, horsetail, valerian, and lemon oil. Conventional growers followed the guidelines of suppliers, and biodynamic growers followed the Demeter principles (4, www.demeter.net). To better compare the two managements, we modified TFI^18^ by subtracting Copper and Sulfur from conventional products and built up a modified index (mTFI) (Fig. 1). The mTFI values and application timing changed significantly in response to high pathogen pressure, as in summer 2016 (Fig. 1). As synthetic fungicides are often systemic, they persist within plants for about 2 weeks. In contrast, all biodynamic preparations sprayed on plants were washed off by rains and were repeated. This may explain (though not completely) the higher mTFI for biodynamic cultures (7, 10, and 13.3) in 2014, 2015, and 2016, respectively (Fig. 1). In addition, biodynamic applications began earlier in the spring and stopped earlier in the summer (Fig. S2), with more Sulfur than conventional cultures (with the exception of 2014). When summed up for all years, mTFI and Sulfur applications were higher in biodynamic management. For copper, beside 2016, the mean quantities applied did not differ in conventional and biodynamic, remaining lower than the norm of 4 kg/hectare/year (Demeter).

### Pathogens loads in vines

**Figure 2:**
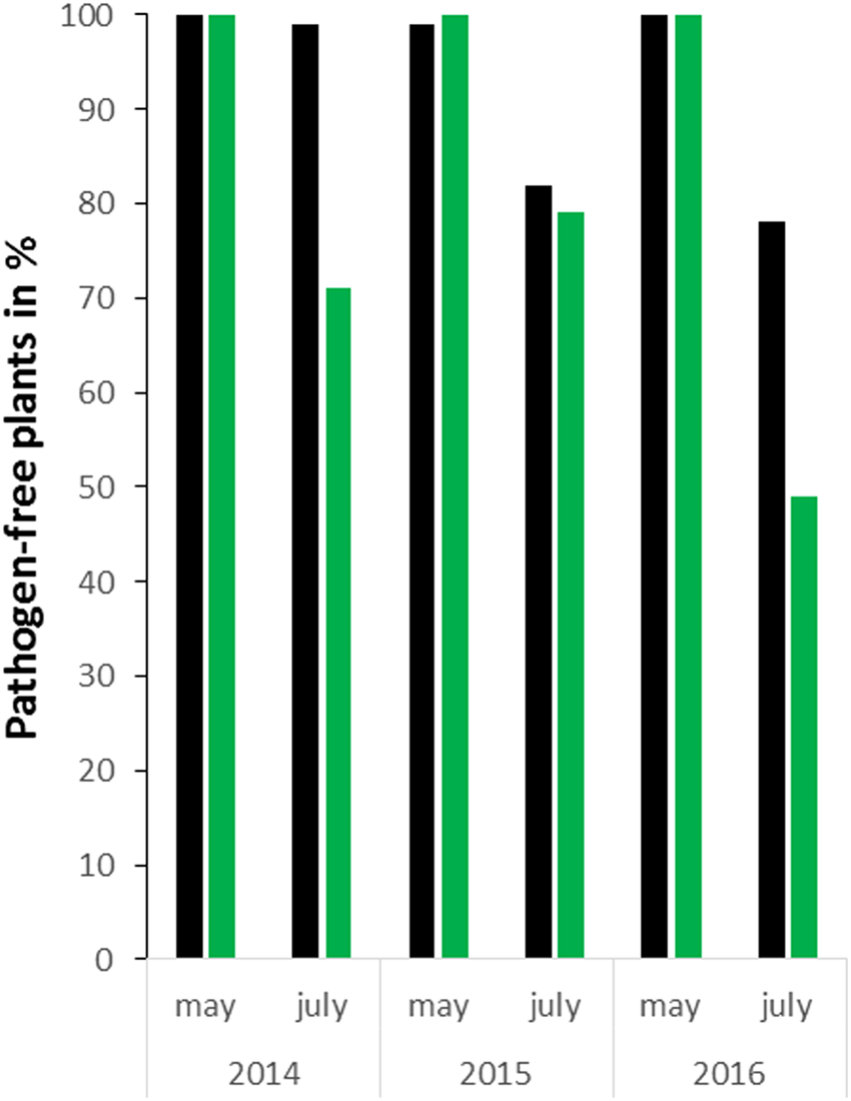
Proportions of pathogen-free *plants in Pinot Noir grown biodynamic and conventional (green and black, respectively). Data from May and July expressed as % of total plants. In total, 2044 pathogen-free samples out of 2648 collected (2014–2016). *Plants free of powdery and downy mildews, of virus (GFLV, GLRaV 1–3 and GVA), after q-RT-PCR and not showing any symptom described in viticulture.

**Figure 3:**
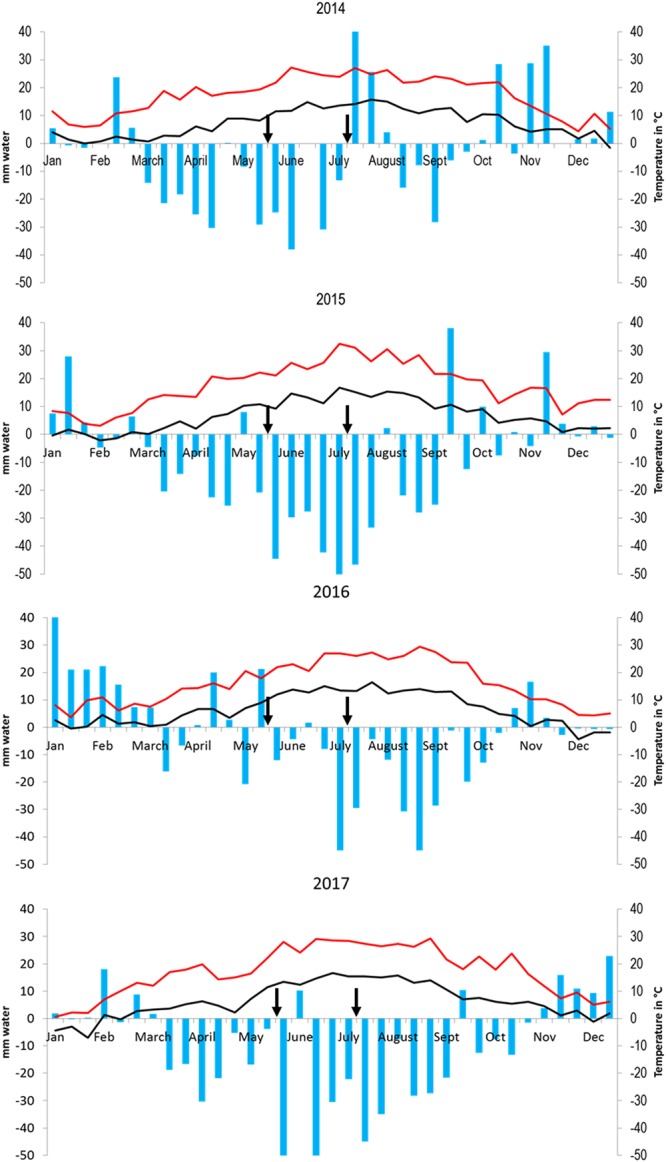
Climatic characteristics. Data were recorded each 10-day period throughout 2014–2017. Differences between rainfalls and evaporation potential ETP-Penman (bars). Negative values are indicative of water stress. Maximal and minimal temperatures (red and black lines, respectively). Dates for leaf-samples harvest used for molecular and biochemical analysis (black arrows).

To evaluate the consequences of different management systems on vines, we examined the forth/fifth leaves from the vine apex, which are the most sensitive to pathogen attack. This developmental stage is associated with a physiological change from sink to source^19^, when the green arms reach the developmental stages H in May and K in July^20^. Molecular analysis by qRT-PCR allowed detection of downy mildew, powdery mildew, as well as viruses such as grapevine fanleaf virus, grapevine leafroll virus and grapevine vitivirus A (GFLV, GLRaV 1–3 and GVA, respectively). Interestingly, 94 to 100% plants were pathogen-free in spring 2014–2016 (Fig. 2). In July 2014–2015, the proportions of pathogen-free plants decreased, notably in biodynamic management. In 2016, facing high pathogen pressure due to humid and warm conditions (Fig. 3), 78% of plants grown conventionally remained pathogen-free and 49% in biodynamic cultures (Fig. 2). In the pooled data (2014–2016), 305 samples were positive for downy mildew and powdery mildew (36 samples with mixed viral and fungus infection not included in calculations). Fungi were more abundant on plants from biodynamic than conventional cultures (18.28% and 7.09%, respectively; independence test χ ^2^ *P* ≤ 0.001). The titers for downy mildew were higher for biodynamic than conventional plants (medians of Δ.ΔCT = 11.60 vs 8.29, Mann-Whitney, *P* ≤ 0.001). For powdery mildew, the Δ.ΔCT medians did not differ between practices (6.46 *vs*. 6.94, Mann-Whitney *P* = 0.21). However, for both managements, none of the harvested leaves showed the visible symptoms described in viticulture, such as a powdery leaf surface due to conidiophores of powdery mildew, an oil-spot leaf response, or associated sporangiophores typical of downy mildew^21^. Thus, both pest managements appeared to hinder the progression of fungal infection *in planta*. The low organic matter and nitrogen contents of the soils (Table S1) may also have reduced pathogen distribution within plots and the multiplication of pathogens on leaves^19,22,23^. With respect to viruses, we observed characteristic symptoms only in autumn^24^. However, molecular analyses of 263 samples collected across 2014–2016 detected infections by one or more of the most frequent grapevine viruses (such as GFLV, GLRaV 1–3 and GVA) already in the spring, suggesting that the viruses were present before the project started. In the case of pathogen threats, whilst both cultivation practices hindered the progress of infections, the wide application of conventional fungicides clearly reduced the frequency of mildews infection of leaves, and the pathogen contents, more than biodynamic management. However, this again is offset by the environmental impact of synthetic pesticides, whereas copper is the only questionable input in biodynamic practice.

### Vines responses to abiotic stress

**Figure 4:**
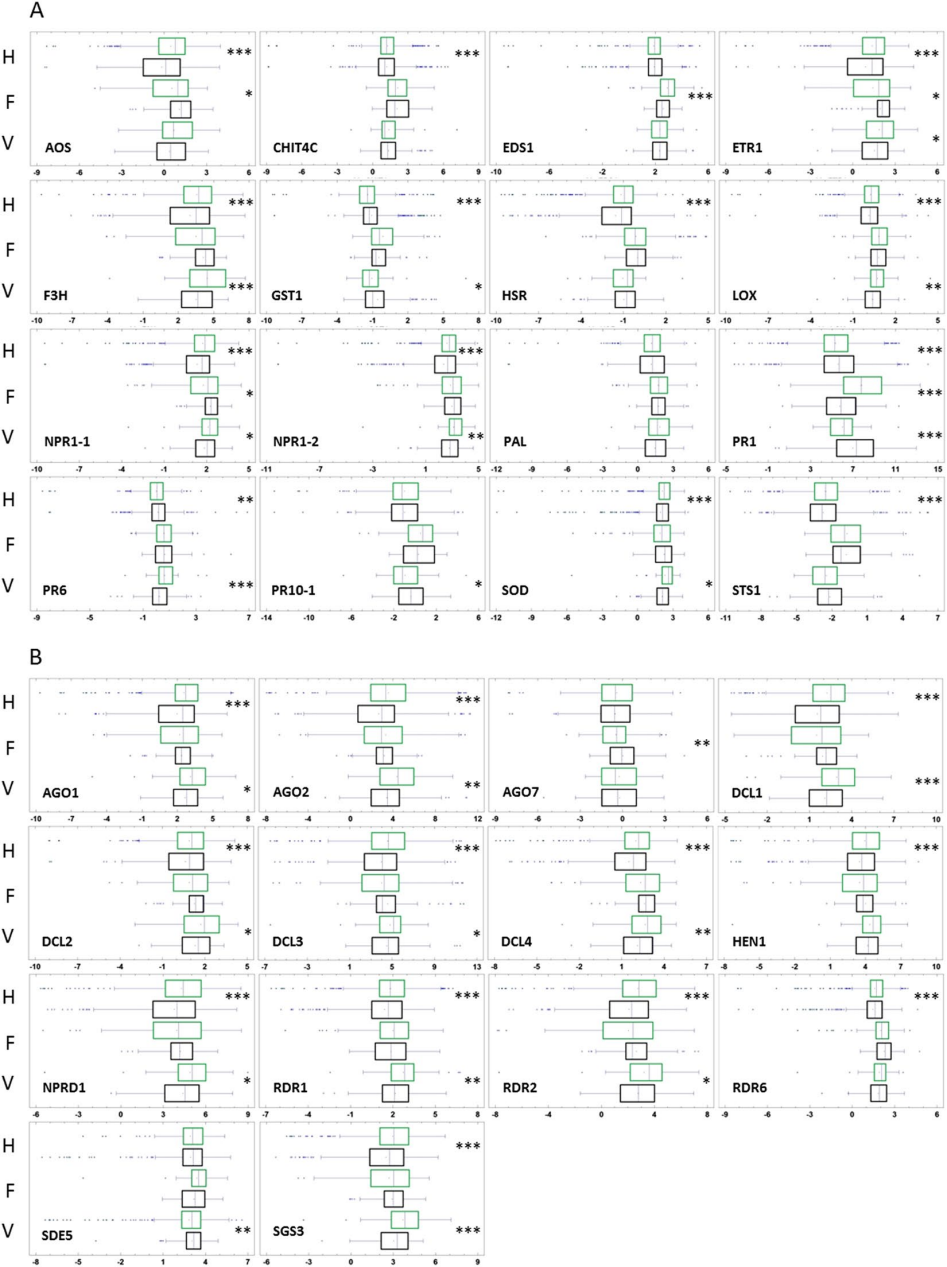
Expression levels of silencing and immunity genes in vines grown conventional and biodynamic. (**A**) Boxplots of Δ.ΔCT of immunity genes calculated for 2014–2016 in leaves pathogen-free n = 2044 (**H**), infected by powdery and downy mildews, (n = 305) (**F**), or by at least one virus (GFLV, GLRaV 1–3 and GVA), (n = 263) (**V**). (**B**) Boxplots of Δ.ΔCT for silencing genes in pathogen-free (**H**), infected by fungi (**F**) and by virus (**V**), green and black boxes for biodynamic and conventional, respectively. Values statistically different, biodynamic/conventional (Mann-Whitney at *P ≤ 0.05, **P ≤ 0.001, ***P ≤ 0.0001).

To characterize vine responses to abiotic stress, we analyzed defense gene mRNA levels in 2044 pathogen-free samples. Variations in mRNA levels of housekeeping genes actin, actin7, GAPDH and UBQ were 15.95%, 13.17%, 8.97% and 6.32%, respectively, and boxplot analyses of normalized Ct values showed the lowest variability of data for GAPDH and UBQ. Therefore, these two genes were chosen as controls for the study. Firstly, mRNA levels of apoplastic amine oxidases (AOS), endochitinase 4 C (CHIT4C), lipase enhanced disease susceptibility (EDS1), ETR1, flavonone 3 hydroxylase (F3H), glutathion S transferase (GST1), HSR, lipoxygenase (LOX), transcriptional activators of the salicylic acid pathway (NPR1-1, NPR1-2), phenyl alanine ammonia lyase (PAL), pathogenesis related proteins (PR1, PR6, PR10-1), superoxyde dismutase (SOD), and stilbene synthase (STS1) were analyzed. These contribute to MAMP-triggered immunity, effector-triggered susceptibility, or effector-triggered immunity^21,25^. Comparing all values from 2014–2016, CHIT4C, ETR1, F3H, STS1, LOX, AOS, NPR1-1, NPR1-2, HSR, SOD transcript levels were higher in pathogen-free samples from biodynamically grown vines (Fig. 4A, H samples). In addition, we analyzed transcript levels of RNA-dependent RNA polymerases (RdR-1, RdR-2, RdR-6), microRNA-generating (Dicer like DCL1), small-interfering-RNA-generating (Dicer like DCL2, DCL3, and DCL4), argonautes (AGO-1, AGO-2, AGO-7), silencing deficient 5 (SDE-5), suppressor of gene silencing 3 (SGS-3), NPRD-1, and HUA enhancer 1 (HEN-1). These contribute to different RNAi pathways involved in the regulation of endogenous and exogenous transcripts levels, defense responses, DNA repair, repair of abiotic and biotic-associated damage, as well as the transgenerational memory of stress^26–30^. Comparing all values from 2014 to 2016, the transcript levels of all RNAi genes except AGO7 and SDE5 were higher in pathogen-free samples from biodynamic vines (Fig. 4B, H samples). These results suggested overall higher levels of gene activation following biodynamic rather than conventional practices.

**Figure 5:**
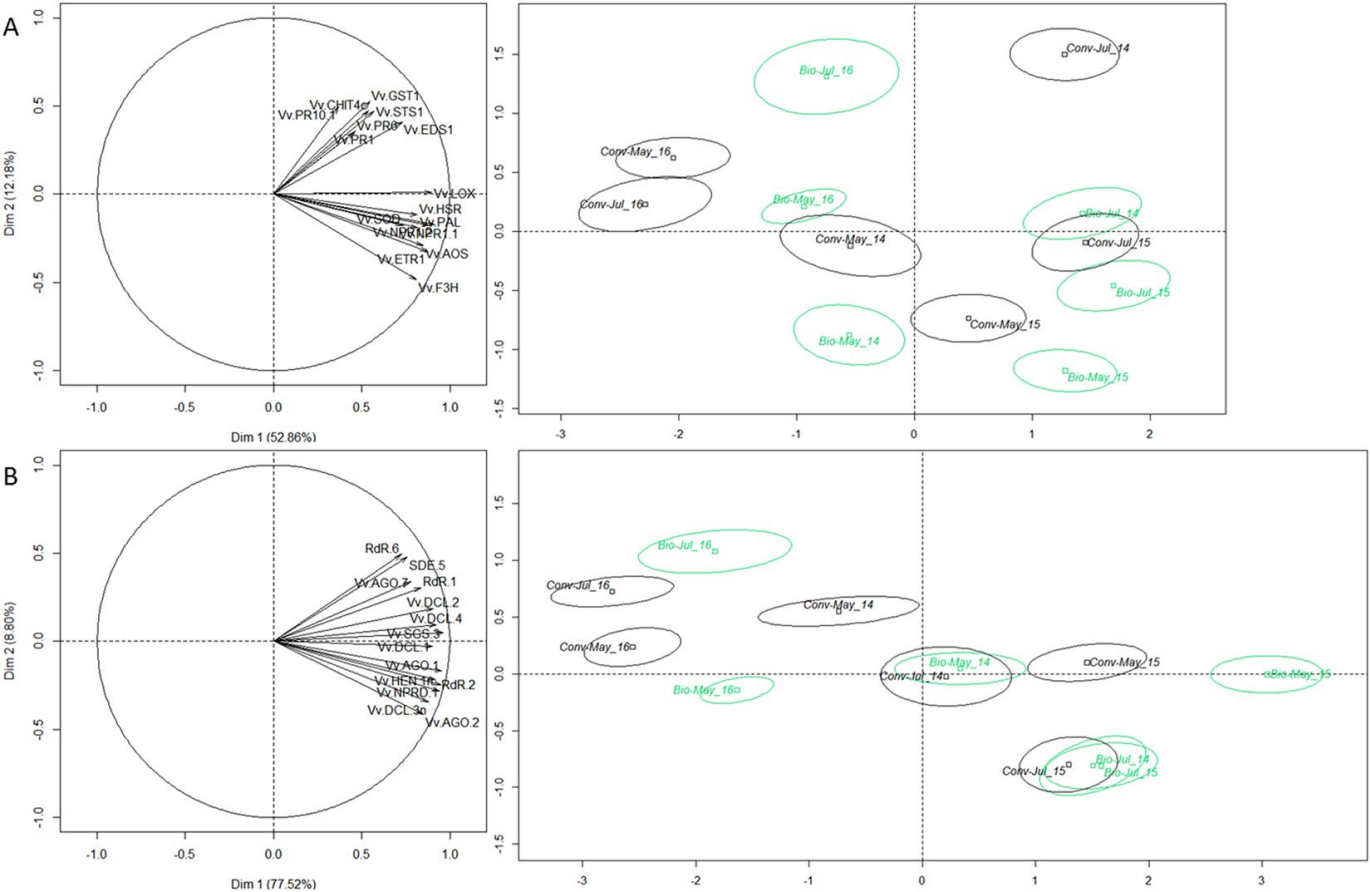
Expression levels of silencing and immunity genes in vines grown conventional and biodynamic. (**A**) PCA analysis for mRNA levels of immunity genes in pathogen-free plants, apoplastic amine oxidases (AOS), Endochitinase 4 C (CHIT4C), Lipase enhanced disease susceptibilty (EDS1), ETR1, Flavonone 3 hydroxylase (F3H), Glutathion S transferase (GST1), HSR, Lipoxygenase (LOX), transcriptional activitors of the sallicic-acid pathway (NPR1-1, NPR1-2), Phenyl alanine ammonia liase (PAL), Pathogenesis related proteins (PR1, PR6, PR10-1), Superoxyde dismutase (SOD), Stilbene synthase (STS1). (**B**) PCA analysis for mRNA levels of silencing genes in pathogen-free plants: RNA dependent RNA polymerases (RdR-1, RdR-2, RdR-6), MicroRNA-generating (Dicer like DCL1), Small-interfering-RNA-generating (Dicer like DCL2, DCL3, and DCL4), Argonautes (AGO-1, AGO-2, AGO-7), silencing deficient 5 (SDE-5), Supressor of gene silencing 3 (SGS-3), NPRD-1 and HUA Enhancer 1 (HEN-1) (1769 leaves collected in May and July, 2014–2016, on plants free of powdery and downy mildews, of virus (GFLV, GLRaV 1–3 and GVA), and not showing any symptom described in viticulture).

We then compared mRNA levels of all RNAi genes for each season with a Principal Component Analysis (PCA on the ranks of Δ.ΔCT). Plants grown biodynamically showed repeatedly distinct levels of expression of RNAi genes between 2014 and 2016 (Fig. 5B). In 2016, the hydric balance (difference between rainfalls and evaporation potential ETP-Penman) was positive (Fig. 3) and thus favorable to vine development. However, by the end of June 2016, dry and warm conditions characteristic of semi-continental/semi-arid climate set in. Thus, we hypothesized that, in conditions of low or very low abiotic stress, and in pathogen-free samples, the activity of the silencing machinery was at a low level (May 2016, Fig. 5B) but increased in response to warmer and drier summer conditions (Fig. 5B). Winegrowers confirmed abiotic stress of vines in summer 2016. Moreover, they pointed out that vines suffered from abiotic stress already in spring 2014, associated with an unfavorable hydric balance and high temperatures (Fig. 3). At the molecular level, the transcript levels of silencing genes were higher in spring 2014 than spring 2016 (Fig. 5B) and a summer-shift was observed, mainly in biodynamic plants. In 2015, when the annual rainfall reached only half of ETP-P and very dry/warm weather persisted from mid-May until the end of July, vines faced severe abiotic threats to the extent that numerous winegrowers across the Alsace region (not participants in the Participative-Action-Research) removed grass with herbicides or by ploughing in order to save their vines. Interestingly, vines in that year exhibited the highest silencing gene transcript levels observed in this study, especially in biodynamic management. Taken together, our data suggest a molecular signature specific to vines grown biodynamically and experiencing a more intense response to abiotic stress, with high expression of silencing and immunity genes (Fig. 5A-B) in pathogen-free leaves. If this reflects enhanced expression of plant defenses then, conversely, the data imply lower plant defense responses in vines subjected to conventional practices. A possible critical difference in plant managements lies in the use of manures and tisanes versus synthetic fungicides. Yet, differences between biodynamic and conventional managements were lower in summer, but also in May and July 2016 (Fig. 5A,B). Importantly, mTFI values as well as amounts of copper and sulfur sprayed were significantly higher in both practices during these seasons (Fig. 1 and Fig. S2). Therefore, smaller differences between management practices in pathogen-free samples, notably in 2016, may be a response to more favorable climatic conditions or/and to excessive spraying impairing plant molecular responses.

### Vines responses to pathogens threats

To compare the outcome of managements in the presence of biotic stress (2014–2016), we analyzed defense gene mRNA levels in pathogen-containing vines grown conventionally or biodynamically. The earlier observations in pathogen-free plants resembled, with higher transcript levels in biodynamic in virus-containing leaves in all RNAi genes but RDR6, AGO7, HEN1. In fungus-containing samples, only AGO7 differed with higher levels in conventional management (Fig. 4B; H, V, F samples). From 2014 to 2016, the comparison between biodynamic and conventional leaves was less clear-cut for immunity genes. Transcript levels of EDS1 and PR1 were higher in biodynamic leaves and ETR1, AOS, NPR1-1 in conventional, in fungus-containing leaf-samples (*n* = 305). Transcript levels of ETR1, F3H, LOX, NPR1-1, NPR1-2, SOD, were higher in biodynamic practices and PR1, PR6, PR10-1, GST1, in conventional practices for virus containing samples (*n* = 263) (Fig. 4A; H, V, F samples). Altogether, the data suggest that silencing genes in vines grown in biodynamic management are more prone to activation by biotic stresses, probably because they are already expressed at higher levels in biodynamic than conventional management in the absence of infection, a phenomenon that resembles priming^31,32^.

**Figure 6:**
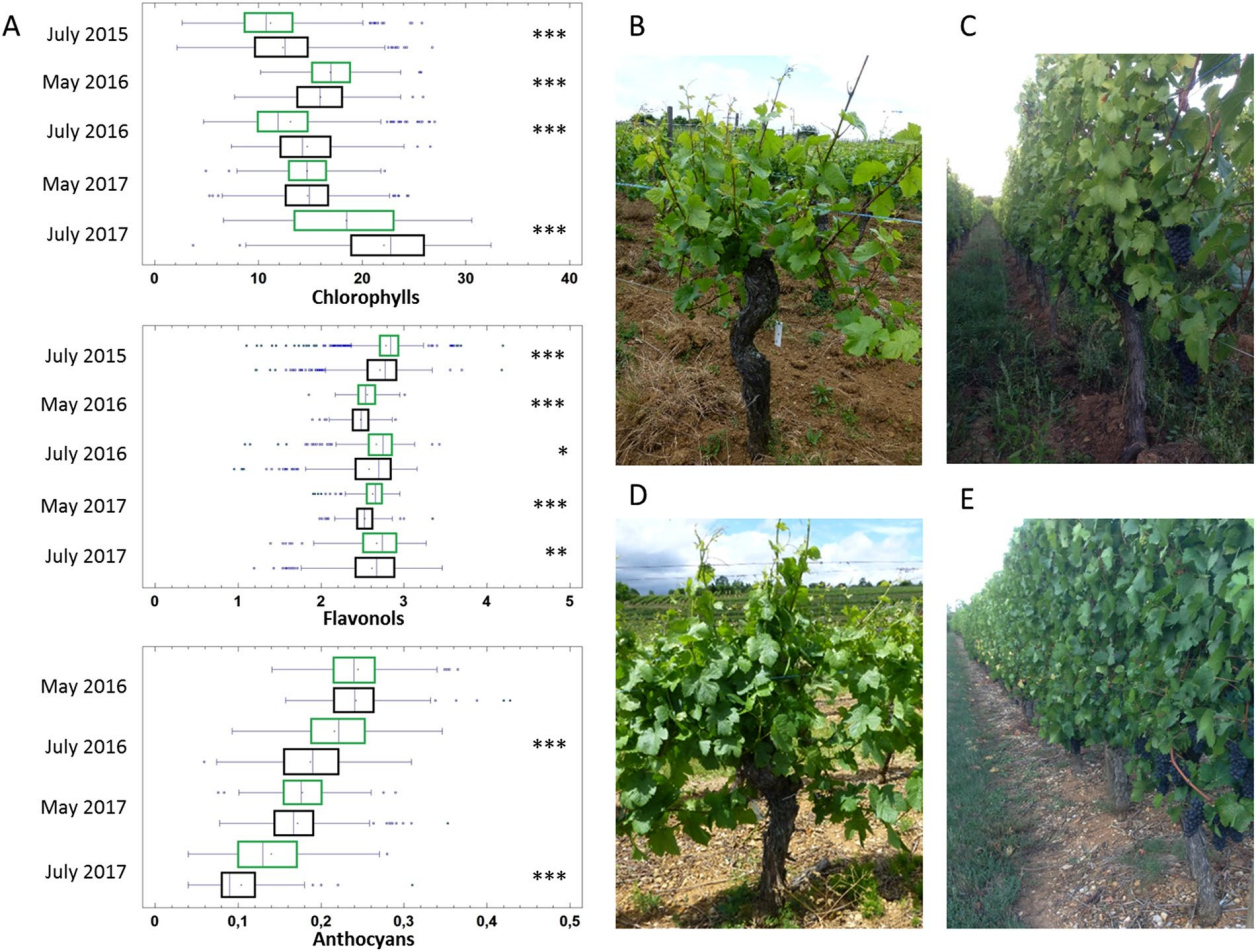
Secondary metabolites contents in leaves of vines grown biodynamic or conventional. (**A**) Chlorophyll, flavonols and Anthocyans. Box plots (in µg/cm^2^ leaf surface) calculated from 3988 leaf samples measured in May and July 2015–2017 (statistically different, according to Man Whitney at *P ≤ 0.05, **P ≤ 0.001, ***P ≤ 0.0001). Biodynamic and conventional (green and black, respectively). (**B–E)** Pinot Noir vines grown biodynamic and conventional in May and September (B-C and D-E, respectively).

During meetings and interviews, conventional growers criticized the yellowish, unhealthy appearance of vines grown biodynamic. In reaction to this observation, we evaluated chlorophyll content as an indicator of plant fitness. In addition, we evaluated flavonols and anthocyans in 3988 leaf samples from all 14-vineyard plots from 2015 to 2017. Both contribute to the capture of and protection from light, as well as to defense reactions^33^. Chlorophyll contents were higher in conventional vines in all samples except May 2016 (Fig. 6). In contrast, flavonols were higher in vines grown biodynamic in all seasons and years. This was also true for anthocyans in July 2016–2017. The results are compatible with the appearance of leaves from biodynamic vines and it is possible that the increase in flavonoids also influenced the chlorophyll-associated green color of the leaves. Interestingly, increase in flavonols and anthocyans may indicate a more effective response to abiotic and biotic stress in biodynamic vines^34^.

Previous research indicates that silicon (“501 preparation”) influence the Arabidopsis transcriptome after fungus infection, and creating a physical barrier on plant leaves^35^. In rice, silicon was reported to alleviate pathogen effects on plant carbon metabolism and cytokinins through priming^36–38^. However, the 501 preparation is applied only once or twice a year, thus other manures from *Equisetum arvense, Salix alba, Achillea millefolium*, or *Valeriana officinalis* may also contribute to elevated defense responses by providing salicylic acid, iron, minerals, and other metabolites. Unfortunately, the constituents and activities of such preparations are poorly documented and they remain a controversy among the vine community. Anyway, causality with plant responses cannot be established firmly here.

### Vines responses to pathogens at the biochemical level

**Figure 7:**
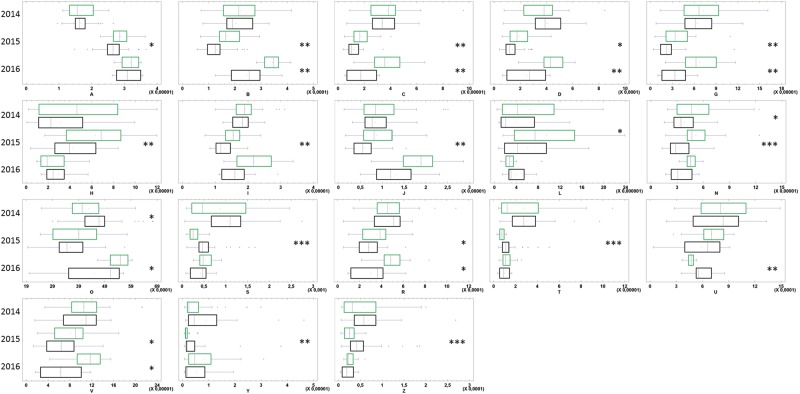
Secondary metabolites contents in leaves of pathogen-free vines grown biodynamic or conventional. Boxplots analysis of 18 secondary metabolites known in grapevine in mg/g leaf from pathogen-free* plants, after UHPLC-MS analysis, n = 142 collected in July 2014–2016. Values statistically different, biodynamic/conventional (green and black, respectively; Mann Whitney at *P ≤ 0.05, **P ≤ 0.001, ***P ≤ 0.0001). *Plants free of powdery and downy mildews, of virus (GFLV, GLRaV 1–3 and GVA), and not showing any symptom described in viticulture. (**A**) 2-[4-(3-Hydroxypropyl)-2-methoxyphenoxy]-1,3-propanediol; (**B**)(−)-Epicatechin; (**C**) Astragalin; (**D**) Quercetin 3-O-rutinoside; (**G**) Astragalin; (**H**) (−)-Epigallocatechin; (+)-Gallocatechin; (**I**) Procyanidin trimer EEC; (**J**) Eriodictyol; (**L**) (−)-Epigallocatechin; (+)-Gallocatechin; (**N**) Procyanidin dimer B1; Procyanidin dimer B2; Procyanidin dimer B3; Procyanidin dimer B4; (**O**) Quercetin 3-glucuronide; (**R**) Isoquercitrin; Quercetin 3-O-glucoside; (**S**) Brevilagin; (**T**) Brevilagin I; **(U**) 2,4,6-Phenanthrenetriol 2-O-b-D-glucoside; (**V**) Delphinidin 3-O-glucoside; (**Y**) (−)-Epigallocatechin 3-O-gallate; (**Z**) Vitilagin.

Pathogen-free leaf samples collected in July of 2014 to 2016, from two plots per practice, were analyzed further by UHPLC-MS. Of the 880-chromatogram peaks, only twenty-seven molecules were already described in vines (Fig. 7 and Fig. S3). The levels of 18 of them increased in response to abiotic stress in a manner resembling the expression of the immunity and silencing genes (Fig. 4), notably with the highest levels in 2015. Of these metabolites, the levels of two were higher in biodynamic than conventional vines in 2014, and 7 in 2016 (Fig. 7). The contents of nine metabolites did not change significantly (Fig. S3). In 2015, the levels of 2-[4-(3-hydroxypropyl)-2-methoxyphenoxy]-1.3-propanediol; (−)-epicatechin; astragalin I; quercetin 3-O-rutinoside; astragalin II; (−)-epigallocatechin; procyanidin trimer EEC; eriodictyol; (+)-gallocatechin; procyanidin dimer (B1/B2/B3 or B4); isoquercitrin/quercetin 3-O-glucoside; delphinidin 3-O-glucoside were higher in biodynamic than in conventional vines (Fig. 7). Interestingly, these flavonols and pro-anthocyanidins have both anti-oxidative and anti-fungal properties^39–42^ and may have enhanced the response to biotic threats, especially in biodynamic vines. In addition, our data confirmed the plasticity of Pinot Noir^17^, particularly when cultivated in biodynamic management.

**Figure 8:**
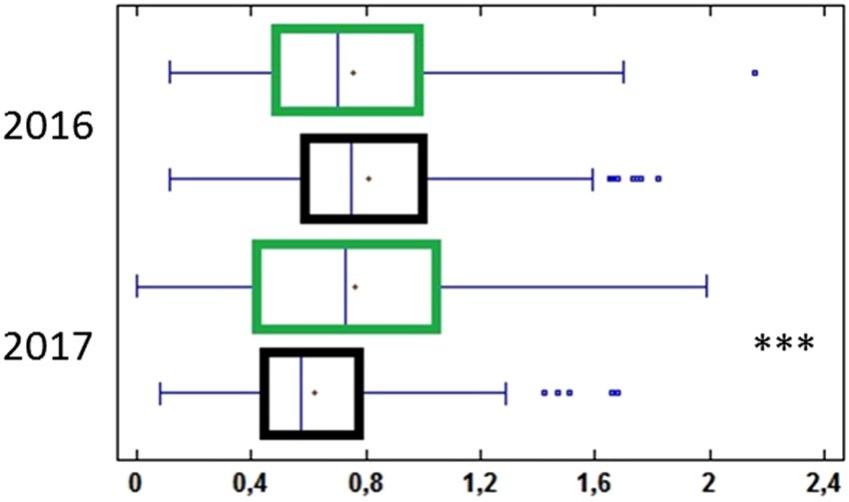
Pruning weight of vines grown biodynamic or conventional, (green and black, respectively). Box plots (in kg/plant) calculated from 1120 data from 40 plants in each of the 14 vine plots measured after falls 2016–2017. (Statistically different, according to Mann Whitney ***P ≤ 0.0001).

Given the enhanced responses of vines grown biodynamic, the question arose of their energetic cost. As yield is primarily determined by management decisions regarding thinning, pruning weights may better reflect overall energy storage of vines. The 1120 data points from 14 plots of mean pruning weights showed no difference between biodynamic and conventional vines in 2016, a favorable year for plant growth (Fig. 8). In 2017, when vines faced intense drought, as in 2015 (Fig. 3), pruning weights of vines grown conventional decreased dramatically, whereas the values remained stable in biodynamic management (Fig. 8; Mann Whitney, *P* ≤ 0.0001). Thus, the enhanced responses to threats in vines grown under biodynamic management were not detrimental to biomass accumulation. On the contrary, it seems that increased resistance to intense climatic stress is associated with maintenance of plant reserves, which contribute significantly to flowering and the grape yield of the following millesimal^43^.

Holistic studies are hampered by the complexity of the interactions between plants, the environment and human practices and reasoning. Biodynamic cultivation still relies on many unvalidated and unrecognized assertions^9^, which hinder acceptance of this form of vineyard management by winegrowers. As a consequence, dialogue between the biodynamic and conventional worlds is still limited. Here we show that involving all of the actors in Participative-Action-Research may contribute resolving the disagreements, firstly by co-constructing questions. Subsequently, all participants were involved in collective reasoning^13,14,44,45^ in a workshop where a consensus was build up from raw data. This led to the main conclusion of this paper. Beyond, the group prioritized its further questioning. Namely, instead of looking for causality of ‘biodynamic management-specific’ plant response to stress at first, the question of ‘when’ such properties are acquired by plants upon change from conventional to biodynamic management was chosen.

## Conclusion

The Participative-Action-Research conducted on this territory lacked winegrowers in organic management. Therefore, we cannot exclude that conclusions raised for biodynamic management would not apply to organic, neither did we resolve fully the controversy about biodynamic practices. However, overall, his project unlocked disagreements between stakeholders by shedding light on unexpected diversity within conventional and biodynamic management, and by characterizing a “biodynamic management-specific” elevated response to climatic and pathogen threats. This suggests that sharing expertise, within a scientific frame, may diminish management intensities, and ultimately, lower environmental and human health impacts of viticulture.

## Material and Methods

### Climatic characteristics

Climate data were recorded for 10-day-periods throughout 2014–2016 and pooled from the ‘climatic database’ (https://intranet.inra.fr/climatik_v2/ClimatikGwt.html?ticket=ST-940746-pH1Dmj3mkgYNnUzacumC-cas). Differences between rainfall and evaporation potential ETP-Penman are shown in mm water (https://en.wikipedia.org/wiki/Penman%E2%80%93Monteith_equation).

### Vineyards plots

Plots were selected so that they all had the same rootstock, planting density and pruning method, upon proposal of winegrowers. The complete trial consisted of 8 vine plots cultivated conventionally and 6 following biodynamic practices, all for more than 20 years. The plots were planted with Pinot Noir vines grafted on the SO4 rootstock at a density of 4,500 plants/hectare. Winter pruning followed the ‘double-Guyot’ guidelines with 1-2 arms (depending on the vigor of the plant) bearing 8–10 latent buds per arm. Summer ‘green pruning’ was conducted to limit vegetative development to c.a. 1 m^2^ foliage/kg bunches, *i.e*. within the wired-frame characteristic of ‘Alsace’ and according to AOC viticulture guidelines. In each plot, after exclusion of plants from the two first border rows and the first and last five plants of each row, 4 blocks of 10 plants were defined. These blocks were positioned as far from each other, with at least 10 plants between 2 blocks, when situated within the same row, and with at least one row distance between two blocks-containing rows.

### Characterization of soils

All data were collected once, in spring 2015. Penetrometer assays were conducted in the 4 blocks of each vine plot (2 measurements/block) with penetrometer (Penetrometre compactometre sol à cone statique: 6120, SPECTRUM Technologies). The first horizon ended when the pressure gauge for penetration reached 200 psi, the second horizon when it reached 300 psi, and the third horizon when a lower pressure was found beyond the second-horizon endpoint. Analysis of structure and physical and chemical composition was carried out on soil samples (c.a. 250 ml) collected in the 0–30 cm horizon in two places in each block. Then all samples were mixed to reach a single sample per vine plot. The following were measured: pH (NF ISO 10390; extraction with 1/5 distilled water); organic matter (sulfochromic method followed by colorimetric assay, NF ISO 14235); nitrogen dosage (extraction with 1 M KCl followed by colorimetric assay; NF ISO 14256-2); phosphorus, potassium, magnesium, calcium, iron, zinc, manganese (10 g soil + 50 ml distilled water, followed by paper filtration and estimation by atomic spectrophotometry (except phosphorus by colorimetric assay) according to BIPEA (https://www.bipea.org/fr/).

### Management and treatments

For biodynamic management, soil and plants were sprayed with the preparations 500 (cow manure), 501 (finely ground silica), 504 (stinging nettle shoots, Urtica dioica. L); and on plants only horsetail (Equisetum arvense L.); 507 (valerian flowers extract, Valeriana officinalis L.); willow (Salix alba L.), fresh barrel compost fermented with yarrow blossoms, valerian flower extracts, and lemon oil. Solids were sprayed at ca. 4–20 g/hectare and liquids ca. 2–10 ml, according to the DEMETER guide. The fungicides sprayed on plants in conventional practices were: CABRIO TOP, CANTUS, CYFLODIUM, DIAZOLE TL, ELECTIS, ELECTIS BLEU, EMENDO V, HOGGAR, KENKIO, KESIS, MILDICUT, NATIVO, PANTHEOS, PERGADO MZ PEPITE, PROFILER, SWITCH, TALIUS, VIVANDO, and YSAYO. As many of these compounds are mixtures, we recalculated the dose for each, after subtracting its content in Copper and Sulfur, when appropriate. The final dosage is expressed as a modified treatment frequency index mTFI = product dose used x field surface sprayed/recommended dose x full field surface for either conventional fungicides or biodynamic preparations. This led to specific indicators for Copper and Sulfur and thus allowed a better comparison of conventional and biodynamic managements. The doses are as recommended by the supplier and/or the government for synthetic fungicides on conventional plots or the Biodynamic Guide (www.demeter, 4) for biodynamic composts and preparations. (Neither herbicides nor insecticides were taken under consideration in mTFI calculations).

### Fluorescence measures

Fluorescence was measured on the fourth/fifth leaf from the apex of all 10 plants of each plot (1 measure/plant; 4 blocks/plot) with a ‘DUALEX’ (DX 17748, Force A) device and data transformed into µg/cm^2^ leaf surface according to^46^.

### Leaf sampling

Fourth/fifth-leaves from the apex were collected in May and July (2014–2016), the stage most sensitive to pathogen attack^19^, when the green arms reached the developmental stages H and K^20^ for all 40 plants of each vine plot. Leaves were deep-frozen in liquid nitrogen. Total RNA extraction was performed according to Romon *et al*.^47^.

### Growth conditions of control plants used for molecular analysis

PN162 and PN40024 lines free of all pathogens evaluated in this study were grown *in vitro* under controlled conditions as described in Romon *et al*.^47^, for RNA extraction and qPCR analysis (see below).

### Primers

Primers were designed according to Trouvelot el al^48^ and Chong *et al*.^49^ for immunity genes, for powdery mildew, and for viruses GVA and GLRaV1-3^50–52^. All other primers were designed, optimized for Tm, and adapted to Fluidigm technology, in the course of this study. Amplified products were cloned and sequenced for alignment according to the corresponding genomes of pathogens^48,49^ and Pinot Noir Genome sequence (https://urgi.versailles.inra.fr/Species/Vitis

**Table:** 

Genes	FW primer (5′3′)	RV primer (5′3′)
Vv GST1	CAAGGCTATATCCCCATTTTCTTC	TGCATGGAGGAGGAGTTCGT
Vv SOD	TGCCAGTGGTAAGGCTAAGTTCA	GTGGACCTAATGCAGTGATTGA
Vv HSR	GGACTACCGACATGCACCTG	CCTGGACAATTCTGCCATCT
Vv AOS	GCCTGGCTTAATCACGACAT	CACCTTCGTCCAGAACATGA
Vv LOX	CCCTTCTTGGCATCTCCCTTA	TGTTGTGTCCAGGGTCCATTC
Vv PAL	TCCTCCCGGAAAACAGCTG	TCCTCCAAATGCCTCAAATCA
Vv PR6	AGGGAACAATCGTTACCCAAG	CCGATGGTAGGGACACTGAT
Vv CHIT4c	TCGAATGCGATGGTGGAAA	TCCCCTGTCGAAACACCAAG
Vv PR10.1	CTGTGGTTGACGGAGATGTT	CCCTTAACGTGCTCTTCAGAG
Vv PR1	GGAGTCCATTAGCACTCCTTTG	CATAATTCTGGGCGTAGGCAG
Vv NPR1.1	GACCACAACCGAGCTTCTTGATC	ATAATCTTGGGCTCTTTCCGCATT
Vv NPR1.2	GCAGGAAACAAACAAGGACAGGAT	CAGCCATTGTTGGTGAAGAGATTG
Vv EDS1	CCCTGAATCATCTAGAATTGCGAAT	GTGATTGCTGTAATTGGTTTAGCAG
Vv ETR1	GTTAGGTAGAACTTTGTCTCTGG	TAACAGGATGCTGAATGGGTATGG
Vv STS1	TACGCCAAGAGATTATCACT	CTAAAGAGTCCAAAGCATCT
Vv F3H	ATCGTGGAGGAGCACAAGAT	TGGATGAGGTGTCAGTTCCA
Vv DCL-1	GTACATCCACTTCCTGGATCAC	CAGAATGTCTCTTGACATGAAGC
Vv DCL-2	GCAGGCGACTTATTATCCACCAG	CATGCTCACAGTCATAGTACCCC
Vv DCl-3	CAAGCTGTGAAGGCTGATGGCC	TCAAGGCATCTAATAGGATCTGGGG
Vv DCl-4	GCAAGAATTGGAAAGATTTGTGGC	GACTTTTGTGATCTTCGACGTTCC
Vv RdR-1	CGCCAACTAAGGTCTTGGATC	CACACCAGTTTTGGGGAACTCTAG
Vv RdR-2	GTTGTTTGGGAGGTGGGAAAG	GCATCTTCCTTAGAGATATGGTAC
Vv RdR-6	CATGCTTACTCCTCCCTGAAG	ATCACCCTCTCCCCGAAGACC
Vv HEN-1	GGCCATACCACAGAAGGGTCC	TTCCCGCACGATGGAATTACTG
Vv SDE-5	CTCGTGAAGCAGATGAAGAATC	CCTTTTGAAATGTTTTCCTCGC
Vv AGO-1	CAATTCAGCCTGTCGCTCCCTC	TCCATCACAGCACGGTTCACC
Vv AGO-2	AACGTGAGCAACTTCCTACACC	CTTGTTCACCTTGCCCTCGG
Vv AGO-7	CTAACCAGAACCAGTACCAGAGC	GTTGTTCTGCTTCCATGAAGG
Vv NRPD-1	GCAAGTGCCATCTGGCCTCCTA	CACACGTAGAGCACTGACAAGAAGG
Vv SGS-3	GGATGAGGAGTTGTACAGGAGGG	GGAATGTCGAGCCTTCACTGC
VvActin-1	TGCTATCCTTCGTCTTGACCTTG	GCACTTCTGGACAACGGAATCTC
VvUBQ	GTGGTATTATTGAGCCATCCTT	AACCTCCAATCCAGTCATCTAC
VvActin-7	GACTACCTACAACTCCATCAT	TCATTCTGTCAGCAATACCA
VvGAPDH	TTCCGTGTTCCTACTGTTG	CCTCTGACTCCTCCTTGAT
**Viruses**		
GFLV	CGGGACCACTATGGATTGGAATGA	CGTTCGGTGATATGGAGAGCG
GLRaV1	CTGACCCTATCGCTGCTACTGA	CTTACTCCCATCAACCCAGGTATC
GLRaV2	TATTCTTCATGCCTCTCAGGATCTG	GCTGCGAGTTCTTGTTGACCC
GLRaV3	AAGTGCTCTAGTTAAGGTCAGGAGTGA	CCACCAGTCTCAGTCCGCTATTACC
GVA	CGACCGAACAATGTACCTGAATACTC	CTAGCATTAGGTCCTACTATATCTACC
(Grapevine Fanleaf-virus, Grapevine leaf roll virus 1–3, grapevine Vitivirus A)		
**Fungi**		
*Plasmapora viticola*	TTCGATATATACATGCGAATGGTG	TCCCCAAGGCAAAACATAAC
*Erysiphe necator*	CTTCGGATTTTTGGATCAGA	GGCACGATCATTGGATTCTT
(Downy mildew and powdery mildew)		

) with Vector NTI.

### Total RNA extraction and quantification

Total RNA was extracted with a Nucleospin RNA plant kit (MACHEREY NAGEL) supplemented with 20 mg/ml polyvinylpyrrrolidone 40 and 1% beta-mercaptoethanol in lysis buffer RA1^47^. RNA was quantified with a Nanodrop NP-1000.

### cDNA synthesis and specific target pre-amplification

cDNA was produced from total RNA (100 ng) in a 5-µl reaction mixture using the FLUIDIGM Reverse Transcription Master Mix Kit (FLUIDIGM Corporation, CA, USA) according to the manufacturer’s instructions. An aliquot of 1.25 µl cDNA was pre-amplified in a 5-µl reaction mixture using the FLUIDIGM PreAmp Master Mix Kit (FLUIDIGM Corporation, CA, USA) with a pool of all 41 pairs of gene-specific primers at a final concentration of 50 nM per primer. The PCR conditions were 95 °C for 2 min, followed by 14 cycles at 95 °C (15 s) and 54 °C (4 min). The pre-amplified products were then treated for 30 min at 37 °C in the presence of 4 U/µl exonuclease I (NEW ENGLAND BIOLABS) followed by 15 min at 80 °C for enzyme inactivation. After a fivefold dilution in DNA suspension buffer (TEKNOVA), pre-amplified products were stored at −20 °C until use in quantitative real-time PCR.

### Massive parallel quantitative real-time PCR

Real-time PCR was carried out with the FLUIDIGM BIOMARK HD System using 48.48 Dynamic Array IFCs for Gene Expression according to the manufacturer’s instructions. Briefly, sample mixtures were prepared by mixing 2.7 µl of each diluted pre-amplified product with 3 µl of 2X SsoFast EvaGreen Supermix with Low ROX (BIORAD) and 0.3 µl of 20X DNA Binding Dye Sample Loading Reagent (FLUIDIGM). In parallel, assay mixtures were prepared by mixing 0.6 µl from each 50 µM of mixed forward and reverse primers with 3 µl of 2X Assay Loading Reagent (FLUIDIGM) and 2.4 µl DNA suspension buffer (TEKNOVA). The dynamic array was first primed with control line fluid and then loaded together with sample and assay mixtures using the BIOMARK IFC Controller MX according to the manufacturer’s instructions. The array was then transferred to the BIOMARK HD for PCR at 95 °C for 60 s, followed by 30 cycles at 95 °C for 5 s and 54 °C for 20 s according to the protocol GE Fast 48*48 PCR + Melt v2.pcl. The data were analyzed with real-time PCR analysis software in the BIOMARK HD system using the parameter settings Quality Threshold 0.65, Linear Baseline Correction Method, and Auto (Global) Ct Threshold Method.

### Internal controls and inter-array calibrators for quantitative real-time PCR

Each 48.48 Dynamic Array integrates a total of 36 test samples and 12 control samples. The latter include a no DNA template (NTC), one positive control sample for fungi contamination (mixed strains contaminated by downy and powdery mildews), and four positive control samples for viral contamination (strain P70 contaminated by GLRa-V1 and GVA, strain Y206 contaminated by GLRa-V2, strain PN40024-31 contaminated by GFLV, and strain Y285 contaminated by GLRa-V3). Six additional controls were used as inter-array calibrators for the compensation of signal variation between BIOMARK HD runs. A stock of control cDNA samples was prepared from strains PN162 CIV (i.e. the accession grown in the vineyards) and PN 40024, both grown *in vitro* under controlled conditions^47^, divided into 3-µl aliquots, and stored at −80 °C until further use. Each aliquot was freeze/thawed only once. Before each BIOMARK HD run, both control cDNA samples were pre-amplified with the pool of all 41 pairs of gene specific primers, treated with exonuclease I, and diluted fivefold as for the test samples. The control products were further subjected to serial ½ dilutions in TE buffer pH 8.0 with low EDTA (INVITROGEN) plus 0.25% Tween 20 (THERMO SCIENTIFIC PIERCE). Three distinct dilutions of both inter-array calibrators were loaded on to each 48.48 Dynamic Array at the same position: dilution 1 corresponding to test sample dilutions, dilution 3 corresponding to ¼ of dilution 1, and dilution 5 corresponding to 1/16 of dilution 1 (civ.dil.1, civ.dil.3, civ.dil.5, 40024.dil.1, 40024.dil.3, and 40024.dil.5).

### Data normalization and calculation of delta-delta Ct

For Q-PCR analyses, CT (threshold cycle) values, representing the target transcript abundance in samples, were calculated by the FLUIDIGM Real-time PCR analysis software using default settings. To compensate for technical variations between qPCR runs, the inter-array calibrators (civ.dil.1, civ.dil.3, civ.dil.5, 40024.dil.1, 40024.dil.3 and 40024.dil.5) were used to calculate a calibration factor (CF, geometric mean of inter-array calibrators CT values) for each plate. All CT values on each plate were then multiplied by CF to obtain inter-plate normalized Ct values. Four control genes (actin, actin7, GAPDH and UBQ) were integrated on all plates. For each plate and for each control gene, we calculated a median value from the inter-plate normalized Ct values. These median values were used to associate a coefficient of variation (cv% = 100*standard deviation / mean) with the control genes. The cv% values were 15.95%, 13.17%, 8.97% and 6.32% for actin, actin7, GAPDH and UBQ, respectively. Using boxplot analyses of the normalized Ct values, we showed that data variability was lowest for GAPDH and UBQ. Hence, we used the data of the GAPDH and UBQ control genes to calculate the delta Ct value in all further experiments. For each plate, a geometric mean of the normalized Ct values from the two control genes (CT reference) was calculated. Delta CT values were then calculated for each gene as: ΔCT = CT reference – CT gene. The normalized target amount in the sample was then equal to 2^ΔΔCt^. ΔΔCT = (Ct reference-Ct target) sample – (Ct reference-Ct target) calibrator.

### Cut-Off

Δ.ΔCT values ranged (in semi-log) from 9–15 for samples experimentally infected with virus, 12.91–16.88 for downy mildew, and 4.50–11.39 for powdery mildew. In samples from the vineyards, the values were 0–11.15, after qRT-PCR for virus, 0–16.88 for downy mildew, and 0–10.48 for powdery mildew. All samples analyzed in this study showing a value below the cut-off of 4.5 were considered ‘pathogen-free’. In control samples from plants grown *in vitro* Δ.ΔCTs were 0–2.5 for mildews and 0–2.39 for viruses.

### Sample preparation

Leaves were ground in liquid nitrogen with pestle and mortar and cooled during further preparation. 100-mg portions of ground leaves were accurately weighted and extracted with 800 µl of 80% aqueous acetone (v/v) during ultra-sonication for 45 min. The sample suspension was centrifuged at 12,000 *g* for 5 min and the supernatants collected. The extracts were diluted 1 + 19 (v/v) with H_2_O and Trolox was used as internal standard at 20 µg/ml in each sample. Aliquots of the dilutions (5 μl) were injected into the LC-MS system.

### UPLC/Q-TOFMS analysis

The UPLC analysis was performed on a Waters Acquity UPLC system using an ACQUITY UPLC column BEH Shield RP18 (1.7 µm, 100 × 2.1 mm i.d., WATERS, Saint-Quentin-en-Yvelines, France). The mobile phase, delivered at 0.3 ml/min, consisted of a gradient mixture of water containing 0.1% formic acid (eluent A) and acetonitrile containing 0.1% formic acid (eluent B). The following gradient was used: 0–4 min, 5% B; 4–7 min, 5–15% B; 7–15 min, 15–25% B; 15–16 min, 25–100% B; 16–19 min, 100% B; 19–19.5 min, 100–5% B; 19.5–22 min, 5% B. Detection was at 280 nm for all studied compounds. The LC system was coupled to a micrOTOF-Q II mass spectrometer (BRUKERDALTONIK, Germany). Eluted components were ionized by electrospray ion source (ESI) operating in negative mode, using N_2_ as the instrument gas, with a drying gas temperature of 200 °C at 9 l/min and a nebulizer pressure of 40.6 psi. Set capillary voltage was 4000 V, end plate offset −500 V, collision cell RF 200 Vpp, energy transfer time 120 µs, and pre pulse storage 1 µs. Data were acquired in MS (m/z range of 100–2000). The system was controlled by Hystar chromatography software (BRUKERDALTONIK) and data analysis carried out with Bruker Compass DataAnalysis 4.0 software (BRUKERDALTONIK, Germany). The results are expressed as mg/g Trolox equivalent, a water-soluble synthetic vitamin E derivative 6-hydroxy-2,5,7,8-tetramethylchroman-2-carboxylic acid used as standard.

### Statistical treatment of data

All statistics used « R » (R Core Team, 2017. R: A language and environment for statistical computing. R Foundation for Statistical Computing, Vienna, Austria. URL https://www.R-project.org/) and FACTOMINER package:^52^ An R Package for Multivariate Analysis. Journal of Statistical Software. 25, pp. 1–18. http://www.jstatsoft.org/v25/i01/). As most of the data did not follow a Gaussian distribution, statistical analysis was performed with non-parametric approaches like Mann - Whitney tests or Spearman coefficients for PCA analysis. Final presentations for box-plots used ≪Statgraphics, Centurion XVII≫ (STATPOINT TECHNOLOGIES INC) and FACTOMINER for PCA^53^.

## Electronic supplementary material

supplemental files
