# Supplementary material for: Responses to climatic and pathogen threats differ in biodynamic and conventional vines
Source: Sci Rep. 2018 Nov 15;8:16857. doi: 10.1038/s41598-018-35305-7 (PMC6237997; doi:10.1038/s41598-018-35305-7)
Supplement: Supplementary file 1 — supplemental files [file 41598_2018_35305_MOESM1_ESM.docx]

**Responses to climatic and pathogen threats differ in biodynamic and conventional vines**

Isabelle Soustre-Gacougnolle, Marc Lollier, Carine Schmitt, Mireille Perrin, Estelle Buvens, Jean-François Lallemand, Mélanie Mermet, Mélanie Henaux, Christelle Thibault-Carpentier, Doulaye Dembelé, Damien Steyer, Céline Clayeux, Anne Moneyron, Jean E. Masson.

**Supplemental files**


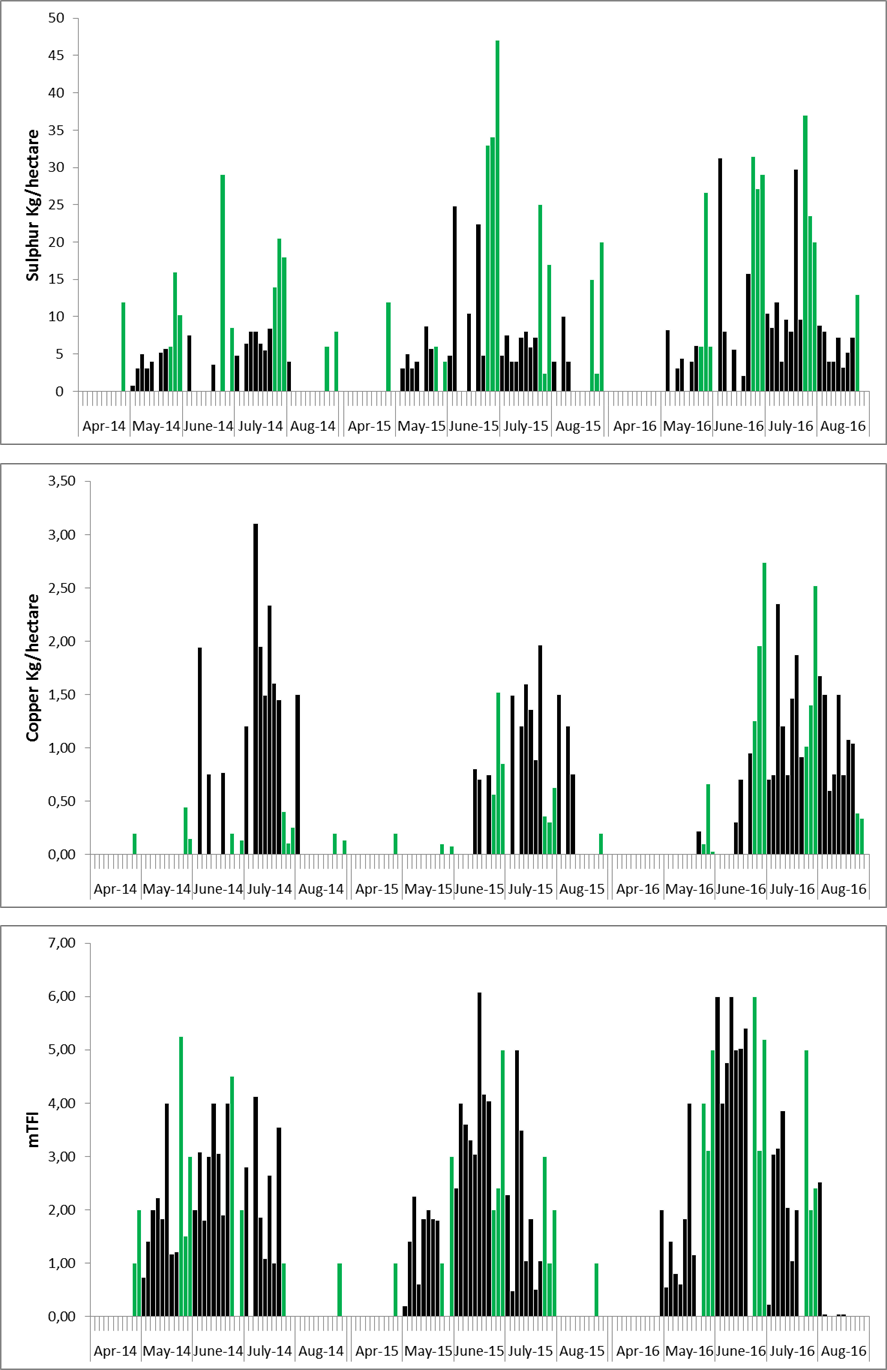


**Figure S1. Characterization of conventional and biodynamic viticulture-practices.** Same legend than Fig. 1 but data represented for each month across 2014-2016.

**
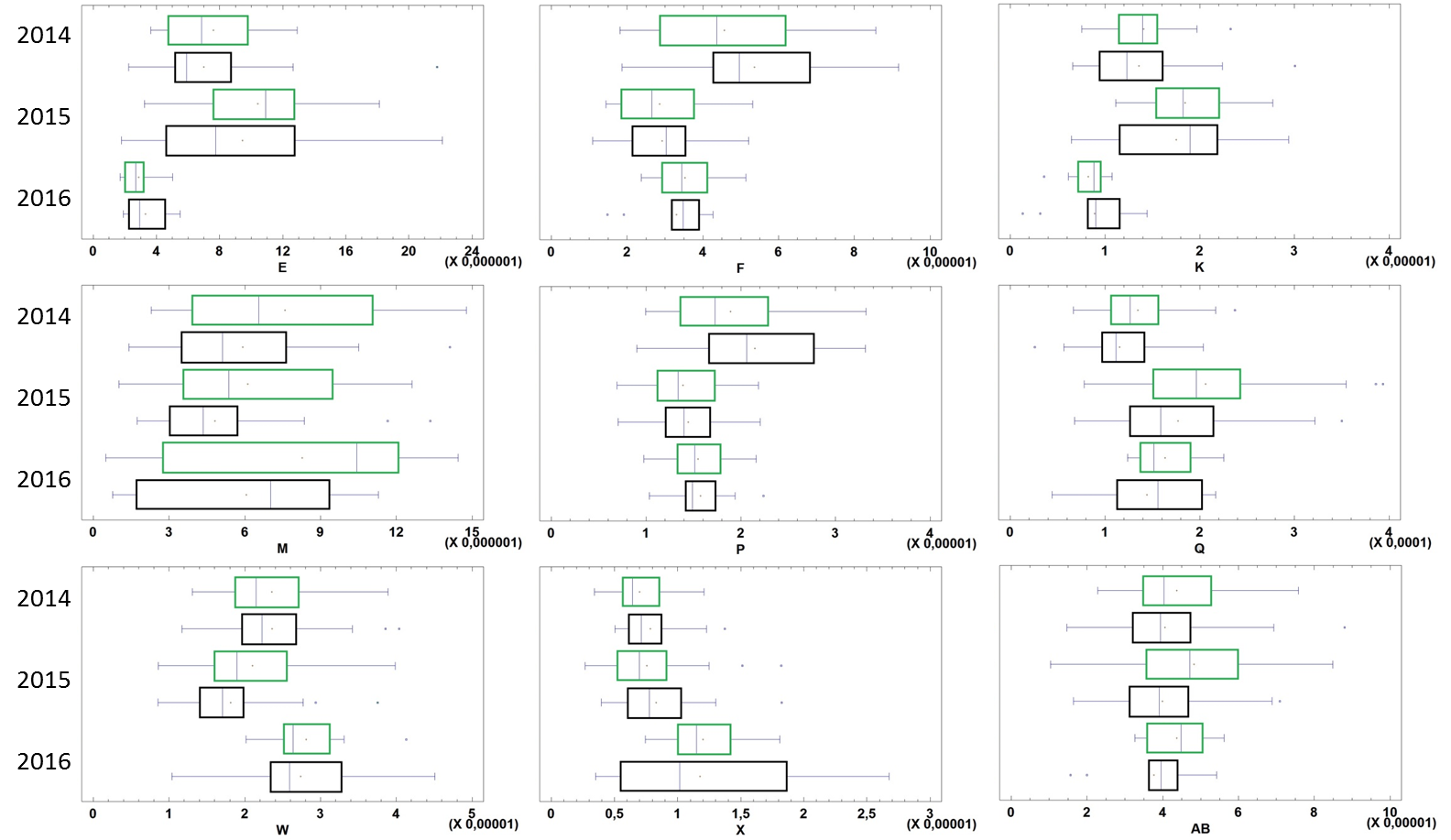
**

**Fig.S2. Boxplots analysis of 9 metabolites known in grapevine** **in mg/g leaf from pathogen-free* plants**, after GC-MS analysis, n=142 collected in July 2014-2016, from vines grown biodynamic and conventional (green and black, respectively). Values not statistically different, biodynamic/conventional. * Plants free of powdery and downy mildews, of virus (GFLV, GLRaV 1-3 and GVA), and not showing any symptom described in viticulture. No clear-cut difference observed for these nine metabolites (on the statistical level). **E**, solariciresinol 4'-O-beta-D-glucoside ; **F**, Castavinol; **K**, Isolariciresinol 4'-O-beta-D-glucoside; **M**, (-)-Epicatechin; **P**, 3-beta-Gentiobiosylglucose; **Q**, cis-Coutaric acid ; **W**, di-caffeic and catechin condensation product ; **X**, sorhamnetin 3-O-glucoside ; **AB**, cis-Coutaric acid.


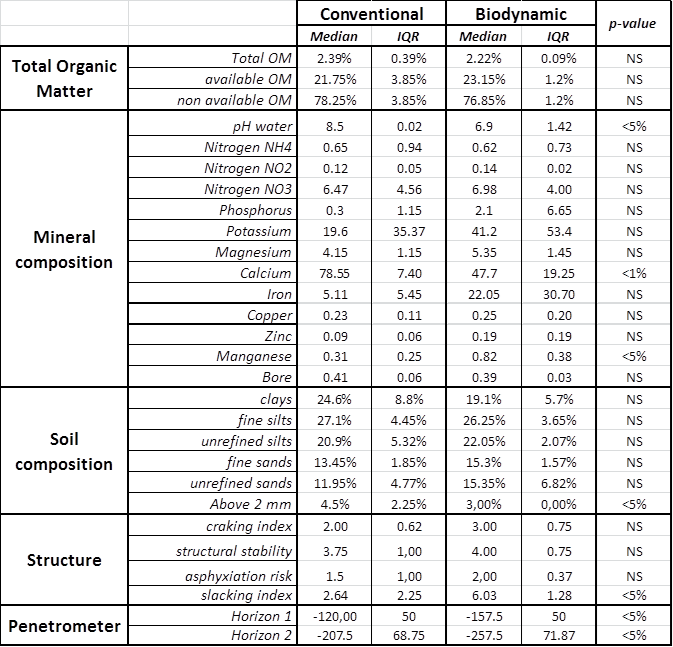


**Table S1: Characterization of soils for vines grown conventional and biodynamic.** Analysis done on 0-30cm soil horizon bulked samples collected from 4 blocks/vine plot. Organic matter (in % total dry matter). pH (in units). Mineral composition in mg/kg soil. Soil composition in %. Structure (in units), and soil horizons in cm.
